# A Systematic Review and Meta-Analysis of Telemonitoring Interventions on Severe COPD Exacerbations

**DOI:** 10.3390/ijerph18136757

**Published:** 2021-06-23

**Authors:** Sujin Jang, Youngmee Kim, Won-Kyung Cho

**Affiliations:** 1 Nursing Department, Seoul National University Hospital, 101, Daehak-ro, Jongno-gu, Seoul 03080, Korea; jsjhb03@naver.com; 2 Red Cross College of Nursing, Chung-Ang University, 84, Heuseok-ro, Dongjak-gu, Seoul 06974, Korea; 3 International Healthcare Center, Department of Pulmonary and Critical Care Medicine, Asan Medical Center, University of Ulsan College of Medicine, 88, Olympic-ro 43-gil, Songpa-gu, Seoul 05505, Korea

**Keywords:** COPD, exacerbation, meta-analysis, systematic review, telemedicine

## Abstract

This systematic review and meta-analysis aimed to provide current evidence regarding the effectiveness of telemonitoring for preventing COPD exacerbations, focusing on severe exacerbations requiring hospitalisation or emergency room (ER) visits. We systematically searched for randomised controlled trials using nine databases from August to September 2020 following the Cochrane Collaboration Guidelines. Of 2159 records identified, 22 studies involving 2906 participants met the inclusion criteria. The participants in 55% and 59% of studies had severe airflow limitations and severe exacerbation histories in the preceding year, respectively. The most commonly telemonitored data were oxygen saturation (91%) and symptoms (73%). A meta-analysis showed that telemonitoring did not reduce the number of admissions (12 studies) but decreased the number of ER visits due to severe exacerbations [7 studies combined, standardised mean difference (SMD) = −0.14; 95% confidence interval (CI): −0.28, −0.01]. Most studies reported no benefit in mortality, quality of life, or cost-effectiveness. All eight studies that surveyed participant satisfaction reported high satisfaction levels. Our review suggested that adding telemonitoring to usual care reduced unnecessary ER visits but was unlikely to prevent hospitalisations due to COPD exacerbations and that telemonitoring was well-accepted by patients with COPD and could be easily integrated into their existing care.

## 1. Introduction

Chronic obstructive pulmonary disease (COPD), which is characterized by chronic irreversible airflow limitation, is a leading cause of mortality and morbidity globally and results in substantial costs and healthcare utilisation [1]. Given the disease burden, researchers have sought new strategies to better care for patients with COPD. 

Telemedicine has drawn attention as a new solution to improve the quality of care for patients with COPD [2,3,4,5,6,7,8,9,10,11,12,13,14]. In general, telemedicine is defined as the “delivery of healthcare services, where patients and providers are separated by a distance” [15]. Notably, to date, the terms telehealth [3,4,5,6,7,8], telemedicine [13], and telemonitoring [9,10,11,14] have been used interchangeably. However, telehealth and telemedicine are broader terms than telemonitoring [16]. Telehealth and telemedicine encompass comprehensive interventions that provide self-management programs, education, consultation, or monitoring over a distance. In contrast, telemonitoring is a term used exclusively for distance monitoring of a patient’s health components as part of a larger chronic care model. In fact, telemonitoring is an essential component of telehealth or telemedicine [16]. 

Numerous studies have tried to apply telemonitoring to COPD to prevent exacerbations [5,9,10,11,12,13,14], reduce healthcare costs [6,8,10,11,14], improve quality of life (QoL) [4,5,10,11,14], establish a self-management program [2], improve physical activity [7], provide education [2], and deliver pulmonary rehabilitation [2,3]. However, the evidence regarding the effectiveness of telemonitoring in COPD management remains inconclusive. 

Despite the lack of robust evidence, interestingly, the vast majority of healthcare providers perceive that telemonitoring will benefit COPD patients [17]. This is probably due to the expected suitability of COPD for telemonitoring applications. COPD is a chronic disease, but patients may experience intermittent acute exacerbations requiring additional treatment. These exacerbations negatively affect the disease course and QoL and are the primary drivers of high medical expenditures in COPD [1]. Therefore, early detection or prevention of exacerbations is paramount to improving clinical outcomes and reducing healthcare costs. Theoretically, if the application of telemonitoring leads to early detection and timely management of exacerbations, it can have a positive impact on reducing morbidity, mortality, and healthcare utilisation in patients with COPD. However, the clinical benefits of telemonitoring for preventing or reducing COPD exacerbations are still controversial and require further elucidation.

We speculated that conflicting results might be related to how a COPD exacerbation is defined, which is usually based on subjective patient symptoms. Some of the exacerbations included in previous studies might not have represented true exacerbations, contributing to the conflicting results. 

With this background, we aimed to provide the most up-to-date evidence regarding the effectiveness of telemonitoring for preventing or reducing COPD exacerbations, particularly focused on severe COPD exacerbations. An exacerbation that requires hospitalisation or an emergency room (ER) visit was defined as a severe exacerbation [1].

## 2. Methods

### 2.1. Protocol and Registration

The protocol for this systematic review is registered in the International Prospective Register of Systematic Reviews (PROSPERO, CRD42020202967). This systematic review and meta-analysis were conducted in accordance with statements and recommendations of the Cochrane Collaboration Guidelines (https://training.cochrane.org/handbooks (accessed on 10 August 2020)) and Preferred Reporting Items for Systematic Reviews and Meta-Analysis (PRISMA) (http://www.prisma-statement.org/PRISMAStatement/ (accessed on 12 August 2020)).

### 2.2. Eligibility Criteria

To determine the eligibility of studies, the participants, intervention, comparison, outcomes, and study design (PICOS) framework was used. The target population (P) was adult patients 18 years and older who were diagnosed with COPD. The intervention (I) was defined as telemonitoring. The comparison (C) was defined as usual COPD care other than a telemonitoring intervention. The outcomes (O) included COPD exacerbations leading to hospitalisation and/or ER visits. The study design (S) included randomised controlled trials (RCTs) along with a control group. Only original research articles written in English with full texts were included. No restrictions on the publication time period were imposed.

### 2.3. Search Strategy and Data Sources

The following nine electronic databases were searched for articles from 10 August 2020 to 10 September 2020: Cochrane Register of Controlled Trials (CENTRAL), Cochrane Review, Cumulative Index to Nursing and Allied Health Literature (CINAHL), EMBASE, PQDT Global, ProQUEST, PubMed, SCOPUS, and Web of Science. Search terms included combinations of text word terms and medical subject headings (MeSH) or EMTREE terms using all possible combinations using Boolean logical operators (AND, OR, and NOT). 

The search keywords were ‘chronic obstructive pulmonary disease’, ‘chronic obstructive airway disease’, ‘chronic obstructive lung disease’, ‘chronic obstructive bronchitis’, ‘telemonitoring’, ‘telehealth’, ‘telemedicine’, ‘telecommunication’, ‘remote consultation’, ‘intervention’, ‘experimental’, ‘trial’, ‘clinical trial’, ‘randomised controlled trial’, ‘randomized controlled trial’, ‘RCT’, and any matched subject or MeSH terms. To avoid missing potentially applicable articles, comprehensive searches were conducted using the keywords above and similar terms. The identified articles were managed using EndNote X9.2 (Thomson Reuters, New York, NY, USA).

### 2.4. Data Extraction

All articles extracted from the nine databases were independently reviewed and selected by two reviewers (investigators S.J. and Y.K.). After excluding duplicate studies, the reviewers chose articles based on the titles and abstracts, including study designs and objectives, according to pre-defined selection criteria. Only original research articles with full texts were included. The review results of the two reviewers were compared, and any disagreements were discussed to reach a consensus. When there were any unresolved discrepancies between reviewers at any stage of the study extraction process, a third reviewer (W.-K.C) was consulted. Finally, all three reviewers (S.J., Y.K., and W.-K.C.) reviewed the full articles again. The study extraction processes were reviewed and verified by all reviewers again.

### 2.5. Risk of Bias Assessment

The risk of bias and methodological study quality were assessed using the Cochrane risk of bias tool, RoB 2.0. Each domain in the evaluation tool was rated as low, high, or of some concern. Two investigators (S.J. and Y.K.) independently evaluated the quality of all the included studies and compared their own assessment results. A third investigator (W.-K.C.) was consulted if there were any discrepancies between the two investigators.

### 2.6. Study Outcomes, Analytic Approach, and Statistical Analysis

The primary outcomes were the number of hospitalisations and/or ER visits due to COPD exacerbations. Hospitalisations and/or ER visits due to non-respiratory causes were not analysed in this review. Exacerbations treated at outpatient clinics were also excluded. The secondary outcomes were QoL, participant satisfaction, anxiety and depression, mortality, and healthcare-related costs. Some studies extended the study duration to the post-telemonitoring period, but this review analysed only the outcomes during telemonitoring in the intervention group [18,19].

A systematic review was first conducted on the included studies. Then, the meta-analysis was also performed to obtain the pooled standardised mean difference (SMD) and 95% confidential interval (CI) if the included studies reported results using the mean and standard deviation (SD) of the primary outcome: number of hospitalisations and/or ER visits. Effect sizes were interpreted as small, medium, and large when they were 0.20, 0.50, and 0.80, respectively [20]. To report the significance of the effect size, 95% CIs were computed. The level of heterogeneity was evaluated using the Q-statistic and inconsistency index (I^2^). Substantial heterogeneity was defined as *p* < 0.05 with a Q statistic or an I^2^ ≥ 50% [21]. A fixed-effects model was used when there was no or low heterogeneity, and a random-effects model was adopted when the level of heterogeneity was high. Funnel plots were generated to assess for possible publication bias. Egger’s test was performed as an additional test to further address publication bias [9,22]. Analyses were performed using R software version 4.0.3 (http://www.r-project.org (accessed on 24 March 2021)).

## 3. Results

### 3.1. Study Selection

**Figure 1:**
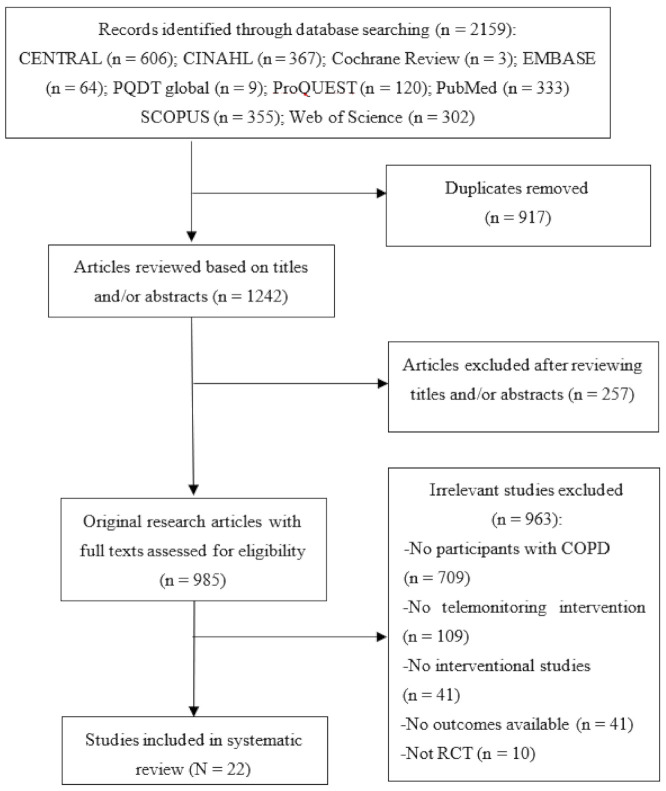
PRISMA flow chart of study selection.

The PRISMA flow diagram in Figure 1 depicts the selection process. The initial literature search yielded 2159 articles. After excluding 917 duplicate articles, 1242 article titles and abstracts were reviewed according to the eligibility criteria. Of these, the full texts of 985 articles were reviewed for eligibility, and 22 RCTs were ultimately included in this systematic review and meta-analysis [18,19,23,24,25,26,27,28,29,30,31,32,33,34,35,36,37,38,39,40,41].

### 3.2. Risk of Bias Assessment

Among the included studies, 91% of the studies (20/22) presented ‘low’ bias in random sequence generation and allocation concealment procedures. Approximately 55% of the studies (12/22) had ‘low’ bias in blinding of participants and personnel. In addition, 86% (19/22) and 77% (17/22) of the studies had ‘low’ bias in blinding of outcome assessment and incomplete outcomes data, respectively. Furthermore, all the studies (22/22) had ‘low’ bias in selection of the reported results.

### 3.3. Systematic Review: Broad Overview and Study Characteristics of All Included Studies

#### 3.3.1. Study Characteristics

Table 1 shows the characteristics of the included studies. A total of 2906 patients with COPD were included in 22 studies. The number of participants varied in each study, and five studies (23%) included ≥100 participants in both the intervention and control groups. In all studies, patients in the control group received the same treatment (defined as usual COPD care) as those in the intervention group, except for telemonitoring. The intervention duration was 2–26 months, and 20 studies (90%) conducted the telemonitoring intervention for ≥6 months. All studies reported the mean participant age, which ranged from 63–81 years. Seventeen studies (77%) reported the percent of predicted forced expiratory volume in 1 s (FEV1), and 12 study participants (55%) had severe airflow limitations according to the Global Initiative for Chronic Obstructive Lung Disease (GOLD) criteria (stage 3) in both the intervention and control groups [1]. Two studies reported the absolute FEV1 (litres, L) and had <1 L of FEV1 [26,28], and all the participants in one study were on home oxygen [19]. These findings indicated poor lung function among the participants. In one study, only participants who had just completed pulmonary rehabilitation were recruited into both the intervention and control groups [18].

The study completion rates in the intervention and control groups were 62–100% and 77–100%, respectively. Thirteen studies (59%) recruited participants with severe exacerbation histories in the preceding year, and four of them recruited participants at the hospital where they were hospitalized for COPD exacerbation [23,31,35,37].

#### 3.3.2. Telemonitoring Intervention

Table 1 presents a summary of the telemonitoring intervention. In most studies, telemonitoring was performed using diverse online platforms connected to devices, such as computers, telephones or mobile phones, that collected and transmitted various parameters, including vital signs, symptoms, oxygen saturation, electrocardiography (ECG) and/or lung function tests, from the telemonitoring devices. The telemonitoring data were reviewed by healthcare personnel. In the event of an abnormal reading or missing scheduled data transmission, the healthcare personnel responded accordingly. We defined this telemonitoring system as ‘standard telemonitoring’. 

Some studies used an Android tablet application [39] or a smartphone application [41] for telemonitoring. In one study, participants directly reported monitoring parameters using a personal digital assistant [36]. One study provided telerehabilitation through video conferencing and telemonitoring [28], and a few studies focused on self-management skill training using telemonitoring systems [19,24,41].

The most common telemonitoring data were oxygen saturation, which was telemonitored in 20 studies (91%), and symptoms, which were telemonitored in 16 studies (73%). Other frequently monitored data were vital signs and spirometry.

#### 3.3.3. Telemonitoring Effects 

Table 2 displays the primary and secondary outcomes of the included studies. All 22 studies examined the number or rate of hospitalisations due to COPD exacerbations, five (23%) of which reported significant reductions with a telemonitoring intervention [23,24,28,30,33]. Thirteen studies evaluated the admission duration, and only two studies (15%) showed a significantly shorter admission duration with telemonitoring [33,42]. Nine studies reported the time to the first admission after initiation of telemonitoring, and only three (33%) reported significant delays [23,33,37]. Thirteen studies evaluated the number of ER visits due to COPD exacerbations; however, a significant reduction was found in only three studies (23%) [24,32,33]. 

Regarding the secondary outcomes, eight studies (36%) investigated cost-effectiveness, and four of them reported significant improvements [19,23,32,42]. Eight studies (36%) assessed participant satisfaction with the telemonitoring intervention using interviews or surveys, and all reported high satisfaction levels [18,19,24,25,27,33,34,42]. Thirteen studies (59%) reported QoL using various tools, and five of them (38%) reported improved QoL [24,32,34,36,39]. Seven studies (32%) that reported mortality showed no change in mortality. 

### 3.4. Meta-Analysis: Telemonitoring Effects on Reducing Severe COPD Exacerbations

#### 3.4.1. Admissions Due to COPD Exacerbations

**Figure 2:**
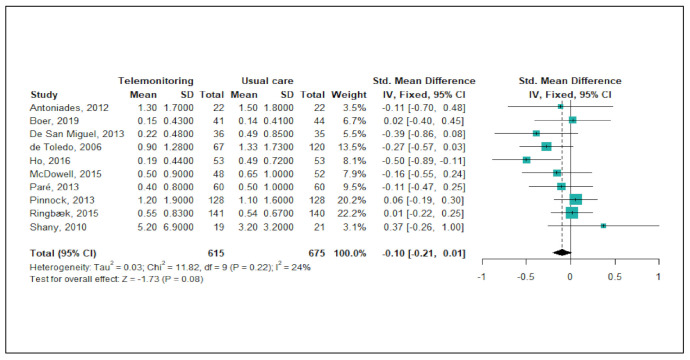
Forest plot of the effectiveness of telemonitoring for decreasing the number of hospitalisations due to severe COPD exacerbations.

Figure 2 shows the results of the meta-analysis of telemonitoring effects on reducing or preventing hospitalisations due to severe COPD exacerbations. Of the 22 studies, a total of 10 studies (45%) were included in this meta-analysis. Since the level of heterogeneity between studies was low (*p* < 0.22; I^2^ = 24%), a fixed-effects model was used. The pooled estimates of the 10 studies showed no significant difference between the telemonitoring and usual care groups with regard to the number of admissions (pooled SMD = −0.10; 95% CI: −0.21, 0.01). A funnel plot and Egger’s test revealed no publication bias (*p* = 0.606). 

#### 3.4.2. ER Visits Due to COPD Exacerbations

**Figure 3:**
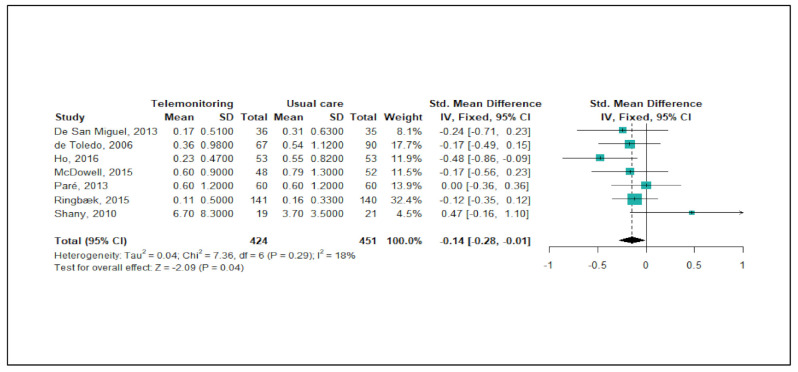
Forest plot for the effectiveness of telemonitoring for decreasing the number of ER visits due to severe COPD exacerbations.

Figure 3 shows the meta-analysis results regarding the effects of telemonitoring on ER visits due to severe COPD exacerbations. Of the 22 studies, a total of seven studies (32%) were eligible for the meta-analysis. As the level of heterogeneity was low across the studies (*p* < 0.29, I^2^ = 18%), a fixed-effects model was adopted. The meta-analysis showed that the telemonitoring intervention effectively reduced the numbers of ER visits (pooled SMD = −0.14 corresponding to a small effect size; 95% CI: −0.28, −0.01). A funnel plot and Egger’s test showed no publication bias (*p* = 0.583).

## 4. Discussion

Numerous studies have been conducted to demonstrate the effectiveness of telemonitoring on reducing or preventing COPD exacerbations. Several systematic reviews have also been published on this topic. However, to date, all of these have reported conflicting results [2,5,8,9,10,11,12,13,14]. With this background, our review aimed to provide the most up-to-date evidence regarding the effectiveness of telemonitoring for preventing or reducing COPD exacerbations. Furthermore, we speculated that conflicting results might be related to how a COPD exacerbation is defined, which is usually based on subjective patient symptoms. Some of the exacerbations included in previous studies might not have represented true exacerbations, contributing to the conflicting results. Therefore, our review focused only on exacerbations that were severe enough to require hospital admission and/or ER visits. 

We reviewed 22 RCTs, which is a considerable number of studies. In the present systematic review and meta-analysis, the methodological quality and risk of bias assessments of the included studies were found to be acceptable. However, there was relatively ‘high’ bias in ‘blinding of participants and personnel’ in the included studies because it might have been difficult for the participants and personnel to remain unaware of the interventions due to the nature of telemonitoring interventions.

Regarding the review findings, we observed significant clinical heterogeneity between trials in terms of the study duration, study population, patient recruitment setting, type of technology employed, and telemonitored parameters. However, most of the studies were conducted in the following format. The intervention durations of most studies were ≥6 months. Most patients with COPD had severe airflow limitations (GOLD stage 3) and a history of severe COPD exacerbation(s) in the preceding year. Telemonitoring was performed using diverse online platforms connected to devices that acted as gateways for data transmission of information measured using telemonitoring devices. The most common telemonitored data were oxygen saturation, symptoms, and vital signs, which were transmitted synchronously or asynchronously with data collection. The information transmitted was usually evaluated by medical professionals so that they could respond to any abnormal or missing data. Some studies implemented additional interventions beyond telemonitoring, such as self-management education or telerehabilitation [19,24,28,41].

Among the studies, 23% reported a decrease in the number of hospitalisations, 15% observed a shortened length of hospitalisation, 33% reported a significant delay in the time to the first admission, and 23% revealed a significant decrease in the number of ER visits with telemonitoring. Therefore, most studies did not report significant benefits of the telemonitoring intervention in relation to preventing severe COPD exacerbations. The meta-analysis further confirmed that telemonitoring did not decrease hospitalisations due to COPD exacerbations. Furthermore, there were no noticeable differences in the study duration, patient characteristics, telemonitoring intervention, or telemonitored parameters between studies that demonstrated telemonitoring benefits and those that did not. However, the meta-analysis showed that telemonitoring significantly reduced the number of ER visits. Intriguingly, all seven studies included in the meta-analysis reported no impact of telemonitoring on decreasing the number of ER visits. 

In sum, our review found that telemonitoring interventions prevented unnecessary ER visits but did not prevent hospitalisations. Based on our review, we conclude that telemonitoring may help to reduce severe COPD exacerbations to some extent. We also evaluated other secondary outcomes, such as QoL, medical costs, mortality, anxiety, and depression, but most studies did not observe a significant effect of telemonitoring on these outcomes. 

The ultimate goal of telemonitoring is to provide a prompt response to abnormal clinical variables so that the patient can receive timely treatment to prevent further deterioration. Given the lack of proven predictors of COPD exacerbations, telemonitoring could act as a great alternative predictor. It is unclear why telemonitoring provided only limited success in preventing severe COPD exacerbations. Given that the information transmitted during telemonitoring was usually evaluated by medical professionals, it was relatively easy to guide patients regarding when to visit the ER, but this intervention was not sufficient to prevent hospitalisations. It is also possible that patients’ own judgements about their health conditions that drive them to seek medical treatment might not be inferior predictors of early exacerbations compared with telemonitored clinical parameters. In other words, patients’ own subjective judgements might be more accurate in predicting COPD exacerbation than small changes in telemonitored data, which can be masked by background variations. In addition, individual heterogeneity, such as comorbidities, disease severity, COPD phenotype, or different history of exacerbations in the preceding year, could have simply contributed to conflicting outcomes in the reviewed studies. For instance, differentiating between a COPD and congestive heart failure exacerbation can sometimes be challenging in hospitalised patients. In addition, standardised treatment provided in clinical practice may also have been highly effective; thus, additional monitoring did not result in significant improvements in COPD care. In all reviewed studies, patients in the control group received the same treatment as those in the intervention group, except for telemonitoring. 

What is the future of telemonitoring in COPD care? In this review, we focused on the effectiveness of telemonitoring for preventing severe COPD exacerbations. However, the possibility that telemonitoring has a positive effect on other clinical outcomes should be assessed to determine the impact of telemonitoring in COPD care. As mentioned earlier, telehealth and telemedicine, which are interchangeable terms, encompass more comprehensive interventions than telemonitoring [16]. For instance, telehealth or telemedicine can involve education, counselling, virtual hospitals, self-management education, or telerehabilitation in addition to telemonitoring [2,3,7,43,44,45,46]. Notably, recent studies addressing the effectiveness of telerehabilitation with telemonitoring for COPD management have reported favourable outcomes [3,7,45,46], and a recent systematic review on self-management integrated with telemonitoring for patients with COPD also showed positive results [2]. A recent study also showed that a subgroup of patients with severe COPD exacerbations could be treated for acute exacerbations at home using telemonitoring [43]. These studies suggest that telemonitoring can be combined with other remote interventions in various application modes. Therefore, further investigation is needed to evaluate the overall role of telemonitoring in COPD care, and a great deal of research on telemonitoring still remains to be performed.

Are there any adverse effects of telemonitoring for patients with COPD? Prior research has reported that telemonitoring can cause alarm fatigue, including false alarms from device errors, leading to worsened QoL [47]. However, the articles reviewed in the present study reported a high level of user satisfaction, high study completion rates, and a lack of reported side effects of telemonitoring. Therefore, our review confirmed that telemonitoring is feasible and can be well-integrated into the usual care for COPD. 

In the past, several systematic reviews have been published with or without meta-analyses to address the effectiveness of telemonitoring for COPD care. Most reviews did not draw definitive conclusions, but some reached favourable conclusions [2,5,8,9,10,11,12,13,48]. A few other reviews concluded that telemonitoring does not provide any benefits for COPD management [4,6,14]. The present review has a few strengths compared with other systematic reviews. Unlike other reviews, our review included only randomised studies. Many previous reviews also did not perform a meta-analysis to evaluate the effectiveness of telemonitoring for COPD care. Our study also included the latest published research as well as the largest number of studies.

However, there are a few limitations to our review. First, our search process was extensive, but some studies may have been missed for various reasons. For instance, we did not include the results of the PROMETE II trial, a multicentre, randomised, 12-month trial of a telemonitoring intervention because it was published in the format of a research letter. This recent trial also did not report a significant benefit of telemonitoring [49]. Second, clinical heterogeneity between trials made it difficult to interpret the study results. Therefore, we may have oversimplified the results of the trials. 

## 5. Conclusions

We conducted a systematic literature review and meta-analysis on the effectiveness of a telemonitoring intervention for reducing or preventing severe COPD exacerbations. Our review suggested that adding telemonitoring to the usual care for COPD may reduce unnecessary ER visits but is unlikely to prevent hospitalizations from COPD exacerbations. This review also found that telemonitoring was well-accepted by patients with COPD and could be easily integrated into their existing care.

## Figures and Tables

**Table 1:** Characteristics of the 22 included studies.

1st Author (Publication Year)	Study Duration (Month)	No. of Participants		Male (%)		Mean Age (Years)		Mean FEV1 (Litre or % of Predicted)		Study Completion Rate (%)		Severe Exacerbation History of the Participants in the Preceding Year	Telemonitoring Intervention	Telemonitoring Data
		IG	CG	IG	CG	IG	CG	IG	CG	IG	CG			
Boer (2019) [41]	12	43	44	58	66	69.3	65.9	53.0%	52.1%	81	93	NI	Used smartphone App & focused on SM	SpO_2_, HR, BP, Symptoms, Spirometry
Walker (2018) [40]	9	154	158	66	66	71	71	50.4%	49.4%	71	77	Yes	ST	Respiratory mechanical impedance. SpO_2_, BP and HR for CHF patient.
Farmer (2017) [39]	12	110	56	62	61	69.8	69.8	47.4%	50.1%	85	86	NI	Used android tablet App with educational video	SpO_2_, HR, Meds, Symptoms
Cordova (2016) [36]	24	39	40	50	27	64	63	31.0%	32.0%	87	83	Yes	Self-Report using PDA	Peak flow rate, BT, Symptoms
Ho (2016) [37]	6	53	53	81	72	81.4	79	62.0%	62.0%	100	100	Yes (H)	ST	BW, SpO_2_, HR, BP, Symptoms
Vianello (2016) [38]	12	230	104	71	73	76	76.5	41.9%	41.9%	79	78	Yes	ST	SpO_2_, HR
McDowell (2015) [34]	6	55	55	42	46	69.8	70.2	45.5%	43.4%	87	95	Yes	ST	SpO_2_, HR, BP, Symptoms
Ringbæk (2015) [35]	6	141	140	39	55	69.8	69.4	34.9%	33.8%	88	89	Yes (H)	ST with video conference	Spirometry, SpO_2_, Symptoms
Bentley (2014) [32]	14	32	31	42.9	28	67.2	65.9	NI	NI	72	80.6	Yes	ST	SpO_2_, HR, BP, Symptoms
Segrelles Calvo (2014) [33]	7	30	30	76	73	75	72.7	38.3%	37.1%	90	87	Yes	ST	BP, SpO_2_, HR, Peak flow rate
De San Miguel (2013) [19]	6	40	40	39	57	71	74	Home O_2_	Home O_2_	90	88	NI	ST	BP, BT, HR, O_2_ flow rate, SpO_2_, Symptoms
Paré (2013) [42]	21.5	60	60	32	32	67.8	68.6	<45.0%	<45.0%	100	100	Yes	ST & focused on SM	Meds, Symptoms
Pedone (2013) [30]	9	50	49	72	63	74.1	75.4	52.5%	55.4%	78	100	NI	ST	SpO_2_, HR, BT, Physical activity
Pinnock (2013) [29]	12	128	128	41	49	69.4	68.4	44.0%	40.0%	82	78	Yes	ST	SpO_2_, Meds, Symptoms
Sorknaes (2013) [31]	26	132	134	40	38	71	72	33.0%	37.0%	92	90	Yes (H)	ST with video conference	HR, SpO_2_, Spirometry, Symptoms
Antoniades (2012) [26]	12	22	22	45	45	70	68	0.91 L	0.66 L	73	91	Yes	ST	Spirometry, BW, BT, BP, SpO_2_, ECG, Meds, Symptoms
Chau (2012) [27]	2	30	23	96	100	73.5	72.2	38.0%	37.7%	73	78	Yes	ST	Meds, SpO_2_, HR, RR
Dinesen (2012) [28]	10	60	51	NI	NI	68	68	0.90L	0.93L	95	94	NI	ST with tele-rehab via video conference	BP, HR, BW, SpO_2_, Spirometry
Lewis (2010) [18]	6	20	20	50	50	67	70	38.0%	40.0%	100	100	NI	ST & post-PR patients only	BT, SpO_2_, Symptoms
Shany (2010) [25]	12	21	21	48	43	72.1	74.2	NI	NI	62	86	NI	ST	BP, Spirometry, ECG, SpO_2_, BW, BT, Symptoms
Koff (2009) [24]	3	20	20	45	50	66.6	65	33.6%	31.1%	95	95	NI	ST & focused on SM	SpO_2_, FEV1, 6MWD, Symptoms
de Toledo (2006) [23]	12	67	90	NI	NI	71	72	42.0%	42.0%	100	100	Yes (H)	ST with video conference	ECG, SpO_2_, Spirometry, BP, HR, Symptoms

**Abbreviations**: 6MWD = 6-min walking distance; BT = body temperature; BW = body weight; CG = control group; CHF = congestive heart failure; FEV1 = forced expiratory volume in one second; H = recruited at the hospital; HR = heart rate; IG = intervention group; Meds = medication adherence; NI = No information; PDA= personal digital assistant; PR = pulmonary rehabilitation; RR = respiration rate; SM = self-management; ST = standard telemonitoring; tele-rehab = telerehabilitation.

**Table 2:** Primary and secondary outcomes of telemonitoring intervention.

1st Author (Year)	Primary Outcomes				Secondary Outcomes (Measurement Tools)
	Adm. no. (Adm. Rate)	Adm. Duration	Time to 1st Adm.	ER Visit No.	
Boer (2019) [41]	NS				Self-efficacy & self-management action: →; Health status (NCSI): →; QoL: (1) CCQ: → & (2) EQ-5D: →
Walker (2018) [40]	(NS)		NS		QoL: EQ-5D: →; Costs: →; Re-Adm: ↓
Farmer (2017) [39]	NS				QoL: (1) SGRQ: → & (2) EQ-5D: ↑; Mortality: →; Nurse contact: ↓
Cordova (2016) [36]	NS (NS)	NS	NS		Dyspnoea ↓ & PEFR: ↑; Mortality: →; QoL: (1) SGRQ: ↑ & (2) SF-36: ↑
Ho (2016) [37]	NS		↑	NS	All-cause admissions and ER visits: ↓
Vianello (2016) [38]	(NS)	NS		NS	Anxiety & Depression (HADS): →; QoL (SF-36): →; Mortality: →; Re-Adm for all-cause and COPD: ↓
McDowell (2015) [34]	NS	NS		NS	Anxiety ↓ & Depression → (HADS); Costs: →; User satisfaction: ↑; QoL: (1) SGRQ: ↑ & (2) EQ-5D: →
Ringbæk (2015) [35]	NS	NS	NS	NS	Mortality: →
Bentley (2014) [32]	↑	↑		↓	QoL (SGRQ): ↑; Costs: ↓ only if admission data were excluded; Mortality: →
Segrelles Calvo (2014) [33]	↓	↓	↑	↓	User satisfaction: ↑
De San Miguel (2013) [19]	NS	NS		NS	Cost: ↓; User satisfaction: ↑; QoL (CRQ): →
Paré (2013) * [42]	NS	↓		NS	Costs: ↓; User satisfaction: ↑
Pedone (2013) [30]	(↓)				Rate of respiratory events: ↓
Pinnock (2013) [29]	NS	NS	NS		Anxiety & Depression (HADS): →; Knowledge (LINQ): →; Self-efficacy (SECD6): →; Mortality: →; QoL (SGRQ): →
Sorknaes (2013) [31]	NS	NS	NS		Mortality: →
Antoniades (2012) [26]	NS	NS			6MWD: →; QoL: (1) CRDQ: → & (2) SF-36: →
Chau (2012) [27]	NS			NS	User satisfaction: ↑; Spirometry: →; QoL (CRQ): →
Dinesen (2012) [28]	(NS)		NS		Cost: →
Lewis (2010) [18]	NS	NS		NS	User satisfaction: ↑; PCP contact ↓
Shany (2010) [25]	NS	NS		NS	Anxiety & Depression (HADS): →; QoL (SGRQ): →; User Satisfaction (Questionnaire): ↑
Koff (2009) [24]	↓			↓	Cost: tend to ↓; User satisfaction: ↑; QoL (SGRQ): ↑
de Toledo (2006) [23]	↓		↑	NS	Cost ↓; Mortality: →; Acceptability to professionals ↑

**Note**: ↑ = Values were significantly increased in the intervention group (IG) compared with the control group (CG); ↓ = Values were significantly decreased in the IG compared with the CG; NS = Values were not significantly different between the IG and CG. * = These studies extended the study duration to the post-telemonitoring period, but this review analysed only the primary outcomes that occurred during telemonitoring in the intervention group. **Abbreviations**: 6MWD = 6-min walking distance; Adm. = admission; CRQ = Chronic Respiratory Disease Questionnaire; EQ-5D = EuroQol 5-dimension questionnaire; ER = emergency room; HADS = Hospital Anxiety and Depression Scale; LINQ = Lung Information Needs Questionnaire; NCSI = Nijmegen Clinical Screening Instrument; PCP = primary care physician; PEFR = peak expiratory flow rate; QoL = quality of life; Re-Adm = readmission rate; SECD6 = Self-Efficacy for Managing Chronic Disease 6-item scale; SF-36 = 36-item short survey; SGRQ = St George’s Respiratory Questionnaire.
